# Cocaine in Dental Surgery

**Published:** 1885-08

**Authors:** H. Th. Hillischer

**Affiliations:** Dentist, of Vienna


### COCAINE IN DENTAL SURGERY.

BY DR. H. TH. HILLISCHER, DENTIST, OF VIENNA.

The experiments which form the subject of the present article were undertaken by the author immediately on reading Dr. Koller's memorable communication respecting the employment of cocaine as an anæsthetic in eye operations, and were prosecuted with increased zeal and hopefulness after he became aware of the success attained by Dr. Ed. Jelinck, in the same field of observation. "Judging," he says, "by what I had learned from others, and had already confirmed by my own observations, I regarded it from the first as useless to attempt the employment of cocaine as an anæsthetic in the extraction of teeth, since it is a well-known fact that none of the substances which have been thus far employed for this purpose are sufficiently penetrating in their action to annul the pain of the operation, a pain caused in part by the laceration of the pulp, but principally by the stretching of the alveolar walls."

I contented myself, therefore, with instituting two separate sets of experiments, in order to ascertain, 1st, the effects of cocaine upon the tissues and constituent parts of the teeth, especially the sensitive dentine and the exposed pulps, and, 2d, its effects, both when applied endermatically to the buccal mucous membrane, and when subcutaneously injected into the same, and afterwards into its underlying osseous structure.

Thus far, I have applied cocaine-hydrochlorate in one hundred and fifty cases: one hundred and ten times to the teeth, and forty times to the gums and bones (in connection with extractions). The first series of experiments had been partially anticipated a couple of years before, at which time I applied quite a number of tests for the purpose of determining whether excessive sensibility of the dentine and of the pulp could not be lowered by means of morphine and chloral hydrate. Not being satisfied, however, with the degree of success obtained in this direction, I refrained from publishing anything upon the subject.

In my latest investigations I made use of these results, applying cocaine-hydrochlorate in two and five per cent. watery solutions, in ten and twenty per cent. solutions of alcohol and water, and in a fifty per cent. alcoholic solution, in the form of powder, and finally, rubbed up into a paste with a trace of glycerine. I also combined cocaine with various proportions of chloral hydrate, viz.; two to one, one-half to one; likewise, with chloral and morphine, in the following proportions:

Cocaine 1, Chloral .05, Morphine ,01.
Cocaine 100, Chloral 5, Morphine 1.
Cocaine 1, Chloral 1, Morphine 1.

A two per cent. solution of cocaine applied to the dentine on pledgets of cotton-wool, or with a camel's hair pencil, obtunds the over-sensitiveness of the tissue in one or two minutes; morphine and chloral hydrate, in a highly concentrated solution, produce far less effect in a much longer time. The more sensitive the dentine the stronger the solution of cocaine which I had to employ. In cases of extremely sensitive dentine in young persons, and also in cachectic subjects, particularly when they were laboring under gastric catarrh, or convalescent from severe febrile diseases, especially at the commencement of the carious process, and also in the resulting affection of the pulp and subsequent complications with peridentitis, I was obliged to place pure cocaine, triturated into a paste with some glycerine, in the carious cavity, and let it remain there ten minutes or longer; in some instances even for twenty-four hours.

In these and in all other cases, the addition to the cocaine paste of a little morph. mur. (best in the proportion of two to one hundred of the cocaine) strikingly increases and hastens the effect.

When the superficial dentine layer is thus anæsthetized, it may be removed by the excavator or the drill; in markedly successful cases the anæsthetizing of the dentine is so complete that the drill may be operated at its full power without causing pain. As soon as sensitive dentine is again reached the application of cocaine is repeated, so much of the carious dentine is removed as can thus be done painlessly, and the procedure is repeated until the cavity has been properly cleansed and prepared for filling.

In this way I have frequently succeeded in cleansing and filling, at a single sitting, and without pain, large carious cavities for children, who, at first, would not allow their teeth to be touched. But even for adults the process of filling will generally lose much of its terrors if cocaine be employed for benumbing the sensitive dentine in place of electricity and the various caustics and narcotics, all of which have failed to effect the object.

Exposed pulps, I found, were considerably less painful after being touched with a five per cent. solution of cocaine (to which I subsequently added morphine); even highly inflamed and swollen pulps I was enabled to cap (by way of experiment), after treatment with the paste of cocaine and glycerine, although, as was foreseen, the narcotic exerted no influence over the course of the pulpitis. Ulceration and destruction of the pulps of the teeth, and incipient peridentitis, always demanded extirpation of the pulp.

Hence, I was led to devise a new method of procedure, in connection with which cocaine will become indispensable in conservative dentistry. If the exposed pulp was already so inflamed as to show little prospect of healing, and in every case as soon as signs of disease of the roots were apparent, I first anæsthetized the pulps superficially with concentrated cocaine-solution, or with the paste, until I could painlessly remove the roof of the pulp-cavity. I then injected, either into the wall of the nerve-canal or directly into the pulp, inserting the point of the Pravaz syringe into the anæsthetized pulp-head, a solution of cocaine, of twenty per cent. or more. In two minutes I was enabled to remove the entire pulp with the nerve-extractor, without giving the patient any pain. Although I have extracted only six teeth in this manner, the full success obtained in all these cases leads me to believe that the process is generally applicable. How great an advance is shown in this method when compared with the use of arsenic, or the barbarous expedient of tearing out the living pulp with the excavator, is evident on the slightest reflection.

The high degree of sensibility in the "cuneiform defects" of the "collum dentis" I could often mitigate considerably by touching them with cocaine solution, but thus far I have not been able to remove it altogether, even by means of the paste.

In my second group of experiments—upon the application of cocaine to the soft parts of the mouth and their bony foundation—the favorable results obtained were generally only of brief duration, as, indeed, was to be expected, in view of this agent's very restricted range of operation, both as to place and time, when locally employed. A burning sensation, proportioned in degree to the amount of alcohol in the solution, is first experienced for a few moments on contact of the latter with the gums. It is succeeded by a feeling of formication in the affected mucous membrane, which is then loosely distended, and gradually becomes insensible, unfortunately, for a short time only. If it is desired to prolong this effect, the cocaine must be frequently rubbed in at short intervals. In large and small non-specific ulcers of the buccal mucous membrane (thrush, aphthæ, etc.), which are often exceedingly painful, the contact of the concentrated solution of cocaine causes a sharp but momentary sensation of burning, followed, after several repetitions of the treatment, by considerable alleviation. For painful extraction wounds, I place in the tooth cavity pledgets moistened with a weak solution of carbolic acid or chinoline, and with a ten per cent. solution of cocaine. In chronic periostitis with great painfulness, I rub in, after the epithelium has been macerated with iodine employed to promote absorption, a concentrated solution of cocaine, or cocaine in substance, and have frequently obtained good results from this endermatic application of the remedy.

Finally, I injected subcutaneously, in two cases of considerable local painfulness, proceeding from bicuspids affected with periostitic exostosis, a syringeful of a five per cent. solution of cocaine, with speedy and permanent success.

